# Challenges ahead

**DOI:** 10.1590/0100-3984.2023.56.1e1-en

**Published:** 2023

**Authors:** Valdair F Muglia

**Affiliations:** 1 M.D., Ph.D., Associate Professor of Radiology, Department of Medical Images, Oncology and Hematology, Ribeirão Preto School of Medicine, University of São Paulo, Ribeirão Preto, SP, Brazil. Email: fmuglia@fmrp.usp.br.

Since its first issue in 1958, *Radiologia Brasileira* (*RB*), the official scientific publication of the Brazilian College of Radiology and Diagnostic Imaging, was established as one of the most important medical imaging journals of Latin America^(1)^.

This successful history is the result of more than six decades of contributions by many people, including the authors, reviewers, editorial office members, and editors. It is undeniable, though, that much of this trajectory can be directly linked to the leadership of Professor Edson Marchiori who was the Editor-in-Chief of *RB* (initially sharing the position with Professor Giovanni Guido Cerri). Despite his busy academic schedule, Professor Marchiori dedicated his heart and soul to ensure the progress of this journal. In the past decade, *RB* experienced unprecedent advances in editorial office organization as well as in the indexation database, as mentioned by him in the last editorial^(2)^.

I’m honored to serve as the next Editor-in-Chief of *RB*. As an academic radiologist, I have been a member of the editorial boards of several of the major international radiological journals, and have served as an associated editor of *RB* for five years. These past experiences prepared me for this new role, and I am looking forward to working with and for the Brazilian radiology community.

First, though, I must express the gratitude of the entire radiological community in Brazil to Professor Marchiori for the tremendous accomplishments during his tenure, which contributed to elevating not only the quality of *RB* but also of radiology practice in Brazil. *RB* articles are reviewed, edited, and published within the planned timeframe, following the highest ethical standards and guaranteeing scientific integrity. Personally, I have no words to express how grateful I am for his patience and willingness to mentor me all these years on the *RB* Editorial Board. These are big shoes to fill!

There are challenges ahead, and now it is time to face them. It is essential to mention two herein.

The first is to complete the indexation of *RB* in the Web of Science database (maintained by Clarivate Analytics). This is arguably the most relevant indexation process for a scientific journal. It is from the Web of Science that the Journal Citation Reports (JCR) is derived, along with the journal’s impact factor^(3)^. The indexation form was filled in late June 2022 and it is currently being evaluated by the Clarivate Analytics team.

Equally important is the need to strengthen the relationship with our target audience, readers and authors. The *RB* intends to be a platform for disseminating high-quality and practical knowledge. For accomplishing this goal, we must provide state-of-art evidence-based articles with a focus on best practices in radiology, e.g. critical reviews and, occasionally, illustrated pictorial articles. At the same time, *RB* is committed to disseminating new science through original articles. New scientific content will continue to be our focus, and manuscripts will be assessed and curated under the highest scientific standards to ensure a fair peer review process. We must emphasize the importance of the contributing authors for a scientific publication, as they are the main supporters of any journal. To meet our authors’ expectations, *RB* is continuously working to improve the editorial process to provide a quick and accurate response to all submissions, ultimately shortening the time interval between the submission and publication.

The peer-review process must be fair and consistently demonstrate high quality to attract the best manuscripts and meet authors’ expectations. Needless to say, this is an enormous job that requires time and dedication. Therefore, I would like to express in this editorial the gratitude of the whole *RB* editorial office to our reviewers, many of whom have selflessly served our society for many years. I would also like to take this opportunity to encourage young researchers who might be interested in joining our group as *RB* reviewers to apply by e-mail (radiologiabrasileira@cbr.org.br) stating their field of interest and including an abbreviated curriculum vitae.

In conclusion, I am committed, along with the entire new Editorial Board of *RB* to maintain the trajectory of a high-quality, peer-reviewed, international, and open-access publication, committed to improving science and the best practices in radiology, always with a patient-driven focus.
